# Serological Association of *Chlamydia pneumoniae* Infection with Age-Related Macular Degeneration: A Systematic Review and Meta-Analysis

**DOI:** 10.1371/journal.pone.0103466

**Published:** 2014-07-25

**Authors:** Xueyu Chen, Vishal Jhanji, Chupeng Chen, Haoyu Chen

**Affiliations:** 1 Department of Emergency Medicine, Shantou Central Hospital, Affiliated Shantou Hospital of Sun Yat-sen University Shantou, Shantou, China; 2 Department of Ophthalmology and Visual Sciences, the Chinese University of Hong Kong, Hong Kong, China; 3 Joint Shantou International Eye Center, Shantou University and the Chinese University of Hong Kong, Shantou, China; 4 Centre for Eye Research Australia, University of Melbourne, Melbourne, Australia; Centre for Eye Research Australia, Australia

## Abstract

**Background:**

We investigated the serological association of *Chlamydia pneumoniae* infection with age-related macular degeneration (AMD).

**Methods:**

A systematic review and meta-analysis was performed. PubMed, Embase, Web of Science and the Association of Research in Vision and Ophthalmology abstracts were searched to identify studies investigating the serological association of *Chlamydia pneumoniae* infection with age-related macular degeneration. The quality of original studies was assessed using the Newcastle-Ottawa scale. Heterogeneity was explored with meta-regression. The odds ratios (ORs) and standardized mean differences (SMD) of *Chlamydia pneumoniae* infection between AMD patients and controls were pooled.

**Results:**

In total, 9 studies met the inclusion criteria using the Newcastle-Ottawa scale scores ranging from 4 to 9. There was heterogeneity among studies due to a difference in the study designs and measurement of exposure to *Chlamydia pneumoniae* infection. The overall OR of *Chlamydia pneumoniae* infection with AMD was 1.11 (95% confidence interval: 0.78–1.57, *P* = 0.56). The overall SMD of antibody titer between AMD and control was 0.43 (95% confidence interval: −0.12 to 0.99, *P* = 0.13).

**Conclusions:**

Evidence from the current published literature suggested no statistically significant association between *Chlamydia pneumoniae* infection and AMD.

## Introduction

Age-related macular degeneration (AMD) is a leading cause of irreversible blindness worldwide. It is characterized by degeneration of photoreceptor/retinal pigment epithelium and, choroidal neovascularization. Several genetic and environmental factors have been found to be associated with the occurrence of AMD [1]. Age is by far the most important risk factor while smoking is another confirmed environmental factor associated with AMD [2]. In 2005, a genome-wide association study discovered that polymorphic variants in the complement factor H (CFH) [3] gene was associated with AMD. Later on, genetic polymorphisms in other factors in the complement pathway were also found to be associated with AMD, such as complement component 3 [4], complement factor B/complement component 2 [5]. Furthermore, serum level of C reactive protein was higher in AMD patients compared to the controls [6]. These results suggested that inflammation plays an important role in pathogenesis of AMD [7].

The hypothesis of association between *Chlamydia pneumoniae* and AMD originated from the role of *Chlamydia pneumoniae* infection in other inflammatory or autoimmune diseases such as arthritis [8] and atherosclerosis [9]. *Chlamydia* infection may trigger the activation of the alternate complement pathway, therefore potentially increasing an individual's risk of developing AMD. Two studies in 2003 found a serological association between AMD and anti-*Chlamydia pneumoniae* antibodies in American [10] and Japanese [11] populations respectively. However, the results reported from ensuing studies could not replicate these findings and consequently the role of *Chlamydia* in the causation of AMD remained controversial.

The purpose of this study was to systematically evaluate the association of *Chlamydia pneumoniae* infection with AMD through a meta-analysis of the published literature.

## Methods

### Inclusion criteria

We included studies that met the following criteria: 1) The study included subjects with early or advanced age-related macular degeneration and non-AMD controls; 2) The study investigated the association of *Chlamydia pneumoniae* infection with AMD; 3) Status of *Chlamydia pneumoniae* infection was determined by serology for measurement of IgG antibody or polymerase chain reaction (PCR) for detection of DNA in the serum. We excluded the following studies: 1) review articles or comments without original data; 2) animal studies or histological studies without data of serum infection; 3) studies investigating the association of *Chlamydia pneumoniae* infection and progression of AMD.

### Literature search

A comprehensive literature search was performed in four databases, Medline in PubMed, Embase, Web of Science and abstracts of the Association of Research in Vision and Ophthalmology (ARVO) annual meetings. Search strategies used were, “*Chlamydia pneumoniae*” or “*Chlamydophila pneumoniae*” and, “macular degeneration”. The references of the included studies or reviews were reviewed as additional references. The literature search was performed for studies published until April 1, 2012 and was further updated up to April 21, 2014. No language limit was applied.

### Screening of articles

All retrieved results were imported into the EndNote software (version X3, Philadelphia, PA). Two independent reviewers (HC and XC) assessed the titles and abstracts of all literature records retrieved from online search. Duplicate records and unrelated articles were excluded after the initial screening. Subsequently, full-texts of relevant records were obtained and a detailed review was performed. For multiple publications using all or part of the same study population, the most recent report was used. A third masked reviewer (VJ) was consulted when required in order to resolve any discrepancy.

### Risk of bias assessment

The risk of bias was assessed by two independent reviewers (HC and XC) using a customized Newcastle-Ottawa scale [12]. The Newcastle-Ottawa scale has been identified [13] as one of the best tools for assessment of observational study designs. For our study, this scale included the following 9 items: 1) adequacy of case definition; 2) representativeness of cases; 3) community controls; 4) definition of controls; 5) comparability of age; 6) comparability of smoking; 7) ascertainment of measurement of *Chlamydia pneumoniae* infection; 8) same method employed for measurement for cases and controls; 9) non-response rate similar for cases and controls. One score was given to each item if the study met the criteria. If the study did not meet the criteria or did not provide clear information, zero score was given to this item. Any disagreement was resolved by discussion with a third reviewer (VJ).

### Data extraction

The data from each included article was extracted by two reviewers independently, including characteristics of study population, diagnostic criteria and phenotype of cases and controls, sample size, and, measurement of *Chlamydia pneumoniae* infection in cases and controls. The measurement of C*hlamydia pneumoniae infection* was expressed as positive/negative, quintile [10] or trisection [14] or mean/standard deviation, in different studies. For quantitative data, we used the lowest quartile as negative. For continuous data, there were two different units used, titers [10], [15] and index [11]. We converted the index to titers by using the titer of 0.2 as a cut-off [16].

The primary outcome of interest in all the studies was association of *Chlamydia* with early or advanced AMD.

### Data synthesis

Stata Software (version 12.0, StataCorp, College Station, TX) was used for the statistical analysis. The comparison of rate of positive *Chlamydia pneumoniae* infection was evaluated using odds ratio (OR) and 95% confidence interval (CI). Continuity correction was applied to the study with zero-cell counts by adding 0.5 to all cells of the study. The comparison of *Chlamydia pneumoniae* infection titers was evaluated using standardized mean difference (SMD) and 95% CI. Heterogeneity among the studies was assessed by the Chi-square test and I^2^ statistic. Meta-regression was used to explore the sources of heterogeneity if a statistically significant heterogeneity was observed (p<0.1 or I^2^>25%) [17], [18]. In meta-regression, the relationship between effect size and covariants including year, study design and method of detection of *Chlamydia pneumoniae* infection were explored. Random effect (DerSimonian and Laird) [19] models were used to pool the ORs or SMDs from individual studies if a statistically significant heterogeneity was found, otherwise, a fixed effect model was used. Funnel plots were used to assess the any publication bias. Egger's test [20] was used to quantify the possibility of publication bias.

## Results

**Figure 1:**
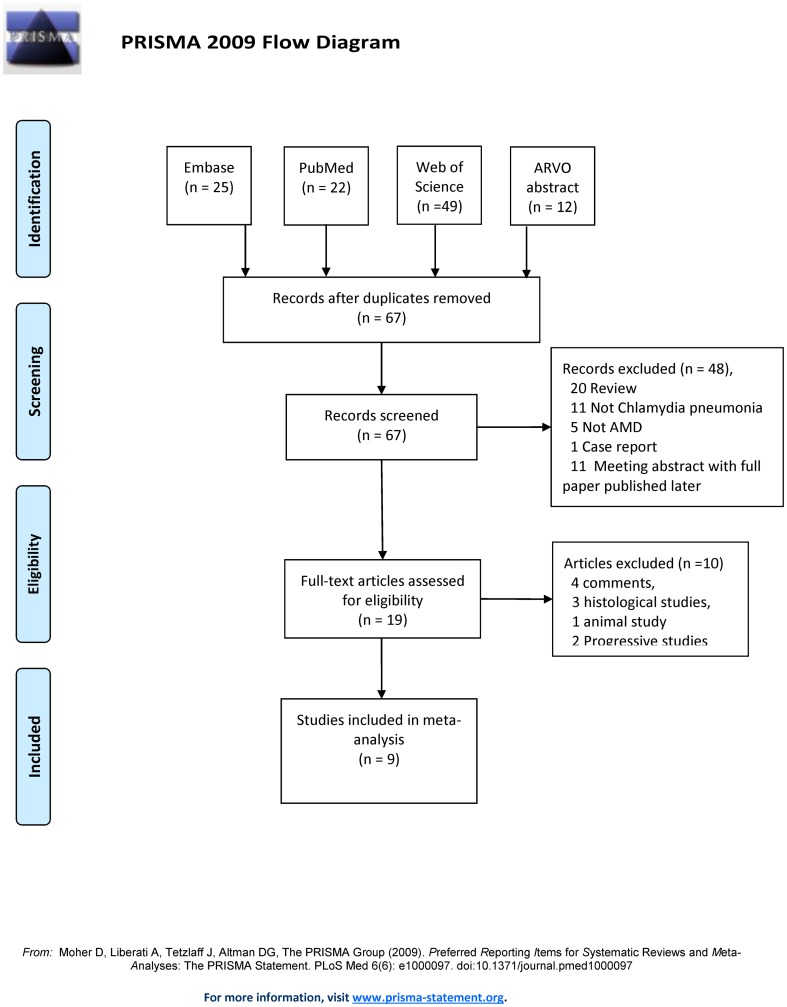
PRISMA flow diagram showing the result of literature screening for meta-analysis.

A total of 108 records were retrieved from online search, including 22 articles from PubMed, 49 from Web of Science, 25 from Embase and 12 from the ARVO annual meeting abstracts. During the initial screening, 41 records identified as ‘duplicate’, and 48 identified as ‘unrelated topic’, were excluded. Full texts of the remaining 19 records were retrieved and reviewed. Four records published as comments or review [21]–[24], 1 animal study [25], 3 histological studies [26]–[28], and 2 studies investigating the association of *Chlamydia pneumoniae* infection with AMD progression but not AMD prevalence [29], [30], were excluded (Figure 1).

The characteristics of the remaining 9 articles [10], [11], [14], [15], [31]–[35] that were included for meta-analysis are shown in table 1. Overall, the study population was from United States of America (n = 4), United Kingdom (n = 1), Austria (n = 1), Australia (n = 1), Japan (n = 1) and Turkey (n = 1). Seven studies [10], [11], [14], [32]–[35] were case control studies and the other 2 were nested case-control studies [15], [31]. The methodology quality scores of the included studies ranged from 4 to 9 (Table 2).

**Table 1:** Characteristics of the Studies Included in the Meta-analysis of Association of *Chlamydia pneumoniae* with Age-related Macular Degeneration.

Author	Population	Design	C.P. Detection	Group	n	Age (years)	Male (%)	Smoking (%)	Type of case/control
Robman [15]	Australian	Nested case–control	ELISA	AMD	197	74.8±7.90	40.6%	51.3%	80.7% early +19.3% advanced AMD
				control	433	72.8±8.10	41.8%	50.1%	Age, smoking matched
Ishida [11]	Japanese	Case-control	ELISA	AMD	27	71.1±6.4	70.4%	NA	choroidal neovascularization
				control	22	69.5±6.5	54.5%	NA	Age matched, >60 years
Kalayoglu [10]	USA	Case-control	ELISA	AMD	25	78.8±6.2	96%	64%	Early + advanced AMD
				control	18	68.7±7.7	100%	33%	>55 years old
Miller [14]	USA	Case-control	ELISA	AMD	83	78.2	55.3%	74.7%	Advanced AMD
				control	67	71.6	51.7%	73.1%	>50 years old
Klein [31]	USA	Nested case-control	ELISA	AMD	188	Matched	40%	16%	75% early +25% advanced AMD
				control	195		41%	17%	Age, smoking matched
Haas [32]	Austrian	Case-control	ELISA	AMD	75	77.0±7.3	45.3%	NA	9.3% early +90.7% advanced AMD
				control	75	76.5±6.5	50.6%	NA	>55 years old
Shen D [35]	USA	Case-control	PCR	AMD	148	79±8	47.3%	NA	Advanced AMD
				control	162	66±11	43.2%	NA	Not mention
Turgut [33]	Turkey	Case-control	ELISA	AMD	40	67.4	62.5%	NA	Early + advanced AMD
				control	20	66.5	50%	NA	Age and sex match
Khandhadia [34]	UK	Case-control	MIF	AMD	199	78.3±8.2	27.1%	NA	26.7% early +73.3% advanced AMD
				control	100	75.6±8.4	44.0%	NA	>50 years old

AMD: age related macular degeneration; n: number; USA: United States of America; UK: United Kingdom; NA: not available; ELISA: Enzyme-linked immunosorbent assay; PCR: Polymerase chain reaction; MIF: Microimmunofluorescence; C.P. *Chlamydia pneumoniae*.

**Table 2:** Assessment of Quality of Methodology of the Studies Using the Newcastle-Ottawa Quality Assessment Scale.

Author	1	2	3	4	5	6	7	8	9	Total score
Robman [15]	1	1	1	1	1	1	1	1	1	9
Ishida [11]	0	1	0	1	1	0	1	1	1	6
Kalayoglu [10]	1	1	0	1	0	0	1	1	1	6
Miller [14]	1	1	0	1	1	0	1	1	1	7
Klein [31]	1	1	1	1	1	1	1	1	1	9
Haas [32]	1	1	0	1	1	0	1	1	1	7
Shen D [35]	1	1	0	1	0	0	1	1	1	6
Turgut [33]	1	1	0	1	1	0	1	1	1	7
Khandhadia [34]	1	0	0	0	0	0	1	1	1	4

‘Y’ denotes ‘yes’ and ‘N’ denotes ‘no’. Quality items: 1) Definition of case; 2) Representativeness of the cases; 3) Selection of Controls; 4) Definition of Controls; 5) Study control for age; 6) Study controls for smoking; 7) Ascertainment of exposure; 8) Same method of ascertainment for cases and controls; 9) Non-response rate.

The inclusion criteria of AMD were clearly defined in all studies except 1 study [11]. However, the criteria were not consistent. Six out of 9 studies [10], [15], [31]–[34] included both early and advanced AMD, 2 studies [14], [35] included only advanced AMD but both geographic atrophy and choroidal neovascularization were included, and 1 study [11] included only choroidal neovascularization. There were 2 population based nested case-control studies [15], [31] and their cases represented the population well. One study selected cases according the genotypes and was therefore likely to have selection bias [34]. The controls were community controls in the two population-based studies [15], [31] but hospital controls in other studies. Definition of controls was clear in all studies except one [34]. The cases and controls were comparable for age in all except three studies [10], [34], [35]. Matching for smoking was reported in two studies [15], [31].

Serology testing was used to measure *Chlamydia pneumoniae* infection exposure in 8 studies, including ELISA in 7 studies [10], [11], [14], [15], [31]–[33], and micro-immunofluorescent assay in one study [34], whereas PCR was used in 1 study [35]. The methodology was same in cases and controls in all studies. The non-response rate was reported in all studies.

**Figure 2:**
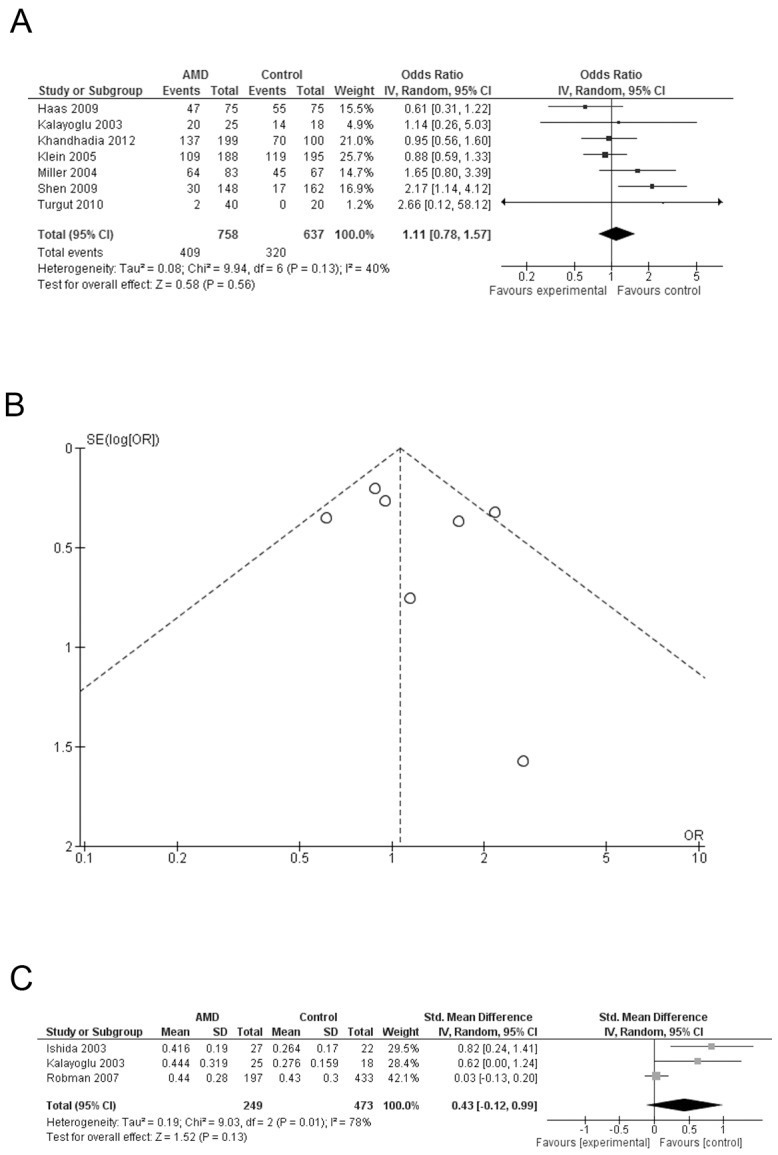
Meta-analysis of the serologic association of with *Chlamydia pneumoniae* and the prevalence of AMD. A: Forest plot comparing the positive rate of *Chlamydia pneumoniae* infection between AMD cases and controls; squares indicate study-specific odds ratio (OR). B: Funnel plots for positive rate of *Chlamydia pneumoniae* infection between AMD cases and controls; C: Forest plot comparing the IgG antibody titers of *Chlamydia pneumoniae* infection between AMD cases and controls; squares indicate study-specific standardized mean difference (SMD). The size of the box is proportional to the weight of the study; horizontal lines indicate 95% confidence interval (CI); diamond indicates summary OR or SMD with its corresponding 95% CI.

Seven out of 9 studies [10], [14], [31]–[35] reported the categorical data (Figure 2A), with a total 758 cases and 637 controls. The individual ORs of having AMD with *Chlamydia pneumoniae* ranged from 0.61 to 2.66. Of these, only one study reported a statistically significant association of *Chlamydia pneumoniae* infection with AMD [35]. The heterogeneity test showed a p value of 0.13, I^2^ = 40%. It was found by meta-regression that the method of measurement might be the source of heterogeneity (I^2^ = 0, p = 0.065), but not the year of study (I^2^ = 48.7%, p = 0.796) or the study design (I^2^ = 41.5%, p = 0.565).

We used a random effect model to pool the individual results. The overall OR was 1.11 (95% CI: 0.78–1.57, *P* = 0.56), suggesting no significant association between *Chlamydia pneumoniae* and AMD. We further performed a sensitivity analysis to exclude the only study using PCR [35]. The results showed I^2^ = 0% and the OR = 0.94 (95% CI: 0.728–1.23, *P* = 0.65). Funnel plot did not suggest any asymmetry (Figure 2B). No significant publication bias was found in Egger's test (p = 0.538).

Three studies [10], [11], [15] reported continuous data (Figure 2C) with a total of 249 cases and 473 controls. Significant SMD of *Chlamydia pneumoniae* antibody titer between AMD cases and controls were reported in two [10], [11] out of these three studies. The heterogeneity test showed *P* = 0.01, I^2^ = 80%. We used a random effect model to pool the individual results. The overall SMD was 0.43 (95% CI: −0.12 to 0.99, *P* = 0.13), suggesting no significant difference of *Chlamydia pneumoniae* antibody titer in serum of AMD cases and controls. It was found by meta-regression that the study design (I^2^ = 0, p = 0.447) was the source of heterogeneity. The two studies reporting a significant difference were case-control studies and the third study with a negative result was a nested case-control study. No significant publication bias was found in Egger's test (p = 0.181).

## Discussion

In this systematic review and meta-analysis, we summarized the current literature on the association of *Chlamydia pneumoniae* infection with AMD. Nine studies with a total of 785 cases and 659 controls were identified. Neither the pooled results of OR of infection positivity rate nor the MD of titers were statistically significant. Therefore, the current literature does not support the association of AMD with *Chlamydia pneumoniae* infection.

Of the nine studies that investigated the association of *Chlamydia pneumoniae* and AMD, statistically significant association was reported in 3 studies [10], [11], [35] whereas the remaining 6 studies did not find a significant association [14], [15], [31]–[34]. The discrepancy in the results reported in different studies may be attributed to a small sample size and/or heterogeneity across the studies. Meta-regression suggested that study design as well as methodology for detection of *Chlamydia pneumoniae* infection were the major causes of heterogeneity. There were 7 case-control studies and the two nested case-control studies. Eight studies measured the IgG antibody [10], [11], [14], [15], [31]–[34] and only one study used the PCR for DNA [35]. The category of AMD was also inconsistent among studies. Some studies included cases with choroidal neovascularization [10], while others included subjects with advanced AMD [35] or both early and advanced AMD [15], [34].

Although our study found no association of *Chlamydia pneumoniae* infection and prevalence of AMD, we believe that it cannot completely exclude the role of *Chlamydia pneumoniae* infection in the pathogenesis of AMD. In a previously reported prospective study in an Australian cohort, the subjects in the upper tertiles of *Chlamydia pneumoniae* antibody titers had two to three times higher risk of AMD progression than those in the lowest tertile. [30] Furthermore, this risk might interact with the genotypes of Complement factor H, which is a major associated gene for AMD [29]. The pathological studies of *Chlamydia pneumoniae* detection in human eyes add further controversy. In a previous study on identification of *Chlamydia pneumoniae* within human choroidal neovascularization tissues, *Chlamydia pneumoniae* was detected in four out of nine AMD choroidal neovascularization tissues by immunohistochemistry and, two out of nine tissues by PCR. *Chlamydia* was not detected in any of the 22 non-AMD specimens [26]. However, in another study, *Chlamydia pneumoniae* DNA was detected in 2/59 (3.3%) AMD samples and in 1/16 (6.2%) control samples. [35] A later PCR report in 13 choroidal neovascularization cases did not detect *Chlamydia pneumoniae* in any of the samples [27]. The relationship between *Chlamydia pneumoniae* and AMD was also investigated in cellular and animal studies. It was reported that *Chlamydia pneumoniae* infection induced production of vascular endothelial growth factor (VEGF) by human macrophages, and, IL-8 and MCP-1 by the retinal pigment epithelium (RPE) cells [26]. In another study, treatment of mouse RPE cells with *Chlamydia pneumoniae* antigen induced expression of IL-6 and VEGF. Vitreous injection of *Chlamydia* pneumoniae antigen increased the size of laser-induced CNV in mice via Toll like receptor-2. [25]

There are some limitations of our study. Firstly, the quality of original research was moderate in some studies. Secondly, pooling results were based on high heterogeneity, with a small number of included studies thereby limiting the exploration of sources of heterogeneity. The characteristics of study subjects were also variable in different studies and the original studies did not report the result of association in each AMD categories. Furthermore, the prevalence of *Chlamydia pneumoniae* may vary according to the geographical location [36]. Although we found that there was no statistically significant association between *Chlamydia pneumoniae* and AMD, this conclusion was based on data from cross-sectional studies. Further prospective or functional studies may be required to further investigate the role of *Chlamydia pneumoniae* in the pathogenesis of AMD.

## Supporting Information

Checklist S1 **PRISMA Checklist.**
(DOC)

## References

[1] Chen Y, Bedell M, Zhang K (2010) Age-related macular degeneration: genetic and environmental factors of disease. Mol Interv 10: 271–281. 21045241 10.1124/mi.10.5.4 PMC3002218

[2] Jager RD, Mieler WF, Miller JW (2008) Age-related macular degeneration. N Engl J Med 358: 2606–2617. 18550876 10.1056/NEJMra0801537

[3] Klein RJ, Zeiss C, Chew EY, Tsai JY, Sackler RS, et al (2005) Complement factor H polymorphism in age-related macular degeneration. Science 308: 385–389. 15761122 10.1126/science.1109557 PMC1512523

[4] Thakkinstian A, McKay GJ, McEvoy M, Chakravarthy U, Chakrabarti S, et al (2011) Systematic review and meta-analysis of the association between complement component 3 and age-related macular degeneration: a HuGE review and meta-analysis. Am J Epidemiol 173: 1365–1379. 21576320 10.1093/aje/kwr025

[5] Thakkinstian A, McEvoy M, Chakravarthy U, Chakrabarti S, McKay GJ, et al (2012) The Association Between Complement Component 2/Complement Factor B Polymorphisms and Age-related Macular Degeneration: A HuGE Review and Meta-Analysis. Am J Epidemiol 176: 361–372. 22869612 10.1093/aje/kws031 PMC6483268

[6] Hong T, Tan AG, Mitchell P, Wang JJ (2011) A review and meta-analysis of the association between C-reactive protein and age-related macular degeneration. Surv Ophthalmol 56: 184–194. 21420705 10.1016/j.survophthal.2010.08.007

[7] Telander DG (2011) Inflammation and age-related macular degeneration (AMD). Semin Ophthalmol 26: 192–197. 21609232 10.3109/08820538.2011.570849

[8] Carter JD, Hudson AP (2010) The evolving story of Chlamydia-induced reactive arthritis. Curr Opin Rheumatol 22: 424–430. 20445454 10.1097/BOR.0b013e32833a43a2

[9] Watson C, Alp NJ (2008) Role of Chlamydia pneumoniae in atherosclerosis. Clin Sci (Lond) 114: 509–531. 18336368 10.1042/CS20070298

[10] Kalayoglu MV, Galvan C, Mahdi OS, Byrne GI, Mansour S (2003) Serological association between Chlamydia pneumoniae infection and age-related macular degeneration. Arch Ophthalmol 121: 478–482. 12695244 10.1001/archopht.121.4.478

[11] Ishida O, Oku H, Ikeda T, Nishimura M, Kawagoe K, et al (2003) Is Chlamydia pneumoniae infection a risk factor for age related macular degeneration? Br J Ophthalmol 87: 523–524. 12714382 10.1136/bjo.87.5.523 PMC1771658

[12] Wells G, Shea B, O'Connell D, Peterson J, Welch V, et al..The Newcastle-Ottawa Scale (NOS) for assessing the quality of nonrandomised studies in meta-analyses .

[13] Deeks JJ, Dinnes J, D'Amico R, Sowden AJ, Sakarovitch C, et al. (2003) Evaluating non-randomised intervention studies. Health Technol Assess 7: : iii-x, 1–173. 10.3310/hta7270 14499048

[14] Miller DM, Espinosa-Heidmann DG, Legra J, Dubovy SR, Suner IJ, et al (2004) The association of prior cytomegalovirus infection with neovascular age-related macular degeneration. Am J Ophthalmol 138: 323–328. 15364212 10.1016/j.ajo.2004.03.018

[15] Robman L, Mahdi OS, Wang JJ, Burlutsky G, Mitchell P, et al (2007) Exposure to Chlamydia pneumoniae infection and age-related macular degeneration: the Blue Mountains Eye Study. Invest Ophthalmol Vis Sci 48: 4007–4011. 17724180 10.1167/iovs.06-1434

[16] Ishida O, Oku H, Ikeda T, Nishimura M, Kawagoe K, et al (2005) Increased Specific Antibody Titers Against Chlamydia pneumoniae in Patients with Age-related Macular Degeneration. Bulletin of the Osaka Medical College 51: 17–22.

[17] Thompson SG (1994) Why sources of heterogeneity in meta-analysis should be investigated. BMJ 309: 1351–1355. 7866085 10.1136/bmj.309.6965.1351 PMC2541868

[18] Thompson SG, Sharp SJ (1999) Explaining heterogeneity in meta-analysis: a comparison of methods. Stat Med 18: 2693–2708. 10521860 10.1002/(sici)1097-0258(19991030)18:20<2693::aid-sim235>3.0.co;2-v

[19] DerSimonian R, Laird N (1986) Meta-analysis in clinical trials. Control Clin Trials 7: 177–188. 3802833 10.1016/0197-2456(86)90046-2

[20] Egger M, Davey Smith G, Schneider M, Minder C (1997) Bias in meta-analysis detected by a simple, graphical test. BMJ 315: 629–634. 9310563 10.1136/bmj.315.7109.629 PMC2127453

[21] Guymer R, Robman L (2007) Chlamydia pneumoniae and age-related macular degeneration: a role in pathogenesis or merely a chance association? Clin Experiment Ophthalmol 35: 89–93. 17300581 10.1111/j.1442-9071.2006.01392.x

[22] Kijlstra A, La Heij E, Hendrikse F (2005) Immunological factors in the pathogenesis and treatment of age-related macular degeneration. Ocul Immunol Inflamm 13: 3–11. 15804763 10.1080/09273940590909185

[23] Patel M, Chan CC (2008) Immunopathological aspects of age-related macular degeneration. Semin Immunopathol 30: 97–110. 18299834 10.1007/s00281-008-0112-9 PMC2441602

[24] Kalayoglu MV, Miller JW (2007) Infection, inflammation and age-related macular degeneration. Clinical and Experimental Ophthalmology 35: 3–4. 17300563 10.1111/j.1442-9071.2007.01432.x

[25] Fujimoto T, Sonoda KH, Hijioka K, Sato K, Takeda A, et al (2010) Choroidal neovascularization enhanced by Chlamydia pneumoniae via Toll-like receptor 2 in the retinal pigment epithelium. Invest Ophthalmol Vis Sci 51: 4694–4702. 20393111 10.1167/iovs.09-4464

[26] Kalayoglu MV, Bula D, Arroyo J, Gragoudas ES, D'Amico D, et al (2005) Identification of Chlamydia pneumoniae within human choroidal neovascular membranes secondary to age-related macular degeneration. Graefes Arch Clin Exp Ophthalmol 243: 1080–1090. 15909160 10.1007/s00417-005-1169-y

[27] Kessler W, Jantos CA, Dreier J, Pavlovic S (2006) Chlamydia pneumoniae is not detectable in subretinal neovascular membranes in the exudative stage of age-related macular degeneration. Acta Ophthalmol Scand 84: 333–337. 16704694 10.1111/j.1600-0420.2005.00591.x

[28] Wolf-Schnurrbusch UE, Hess R, Jordi F, Stuck AK, Sarra GM, et al (2013) Detection of Chlamydia and complement factors in neovascular membranes of patients with age-related macular degeneration. Ocular immunology and inflammation 21: 36–43. 23590149 10.3109/09273948.2012.726393

[29] Baird PN, Robman LD, Richardson AJ, Dimitrov PN, Tikellis G, et al (2008) Gene-environment interaction in progression of AMD: the CFH gene, smoking and exposure to chronic infection. Hum Mol Genet 17: 1299–1305. 18203751 10.1093/hmg/ddn018

[30] Robman L, Mahdi O, McCarty C, Dimitrov P, Tikellis G, et al (2005) Exposure to Chlamydia pneumoniae infection and progression of age-related macular degeneration. Am J Epidemiol 161: 1013–1019. 15901621 10.1093/aje/kwi130

[31] Klein R, Klein BE, Knudtson MD, Wong TY, Shankar A, et al (2005) Systemic markers of inflammation, endothelial dysfunction, and age-related maculopathy. Am J Ophthalmol 140: 35–44. 15939388 10.1016/j.ajo.2005.01.051

[32] Haas P, Steindl K, Schmid-Kubista KE, Aggermann T, Krugluger W, et al (2009) Complement factor H gene polymorphisms and Chlamydia pneumoniae infection in age-related macular degeneration. Eye (Lond) 23: 2228–2232. 19169230 10.1038/eye.2008.422 PMC4853919

[33] Turgut B, Uyar F, Ilhan F, Demir T, Celiker U (2010) Mycoplasma pneumoniae and Chlamydia pneumoniae seropositivity in patients with age-related macular degeneration. J Clin Med Res 2: 85–89. 21811525 10.4021/jocmr2010.03.282w PMC3140884

[34] Khandhadia S, Foster S, Cree A, Griffiths H, Osmond C, et al (2012) Chlamydia infection status, genotype, and age-related macular degeneration. Mol Vis 18: 29–37. 22259222 PMC3258520

[35] Shen D, Tuo J, Patel M, Herzlich AA, Ding X, et al (2009) Chlamydia pneumoniae infection, complement factor H variants and age-related macular degeneration. Br J Ophthalmol 93: 405–408. 18996904 10.1136/bjo.2008.145383 PMC2746818

[36] Blasi F, Tarsia P, Arosio C, Fagetti L, Allegra L (1998) Epidemiology of Chlamydia pneumoniae. Clin Microbiol Infect 4 Suppl 4S1–S6. 11869264

